# The impact of sucralose and neotame on the safety of metal precipitation in electronic cigarettes

**DOI:** 10.3389/fphys.2024.1437042

**Published:** 2024-08-21

**Authors:** Xinyang Yan, Zheng Chen, Xianfang Rong, Zhichao Chen, Guanlin Wu, Zeyi Dong, Yao Fu, Tao Hai

**Affiliations:** Research and Development Center, IMiracle (Shenzhen) Innovation Technology Co., Ltd., Shenzhen, China

**Keywords:** electronic cigarettes, aerosol, neotame, sucralose, heavy metals, cytotoxicity

## Abstract

This study investigated the impact of sweeteners on the release of heavy metals during the heating and atomization processes in electronic cigarettes. Based on a PG/VG base e-liquid with the addition of 2% and 5% neotame or sucralose, we quantitatively analyzed the impact of sweetener content on the levels of heavy metals such as Ni, Cr, and Fe in the e-liquid and aerosol after heating and atomization. Additionally, the heated e-liquid samples were used to culture SH-SY-5Y and Beas-2B cells, and their cytotoxic effects were assessed using the CCK-8 assay. The results indicated that the e-liquid with 5% sucralose had the highest average levels of heavy metals after heating and atomization, particularly nickel (13.36 ± 2.50 mg/kg in the e-liquid and 12,109 ± 3,229 ng/200 puffs in the aerosol), whereas the e-liquid with neotame had significantly lower average heavy metal content in comparison. Additionally, it was measured that the chloride ion concentration in the e-liquid with 5% sucralose reached 191 mg/kg after heating at 200°C for 1 h, indicating that heating sucralose generated chloride ions, Which might corrode metal parts components leading to heavy metal release. Cytotoxicity tests revealed that the base e-liquid without sweeteners exhibited the highest average cell viability after heating, at 64.80% ± 2.84% in SH-SY-5Y cells and 63.24% ± 0.86% in Beas-2B cells. Conversely, the e-liquid variant with 5% sucralose showed a significant reduction in average cell viability, reducing it to 50.74% ± 0.88% in SH-SY-5Y cells and 53.03% ± 0.76% in Beas-2B cells, highlighting its more pronounced cytotoxic effects compared to other tested e-liquids. In conclusion, sucralose in e-liquids should be limited preferably less than 2%, or replaced with neotame, a safer alternative, to minimize health risks.

## 1 Introduction

Electronic cigarettes were device that simulates smoking by producing an aerosol, commonly referred to as vapor, typically containing nicotine, flavorings, and other chemicals, which the user inhaled. As an alternative to traditional tobacco products, electronic cigarettes (e-cigarettes) gained global popularity in recent years (Martins et al., 2022). Compared to conventional cigarettes, e-cigarettes offered a safer method of nicotine delivery, as they did not produce carcinogenic substances such as polycyclic aromatic hydrocarbons, tobacco-specific nitrosamines, and benzene during the atomization process (Breland et al., 2017). This can significantly reduce health risks to smokers, and some national health agencies considered e-cigarettes to be more effective than nicotine replacement therapies in quitting conventional smoking (Szumilas et al., 2022; Toll et al., 2024). The safety of e-cigarettes, a distinct category of consumer electronics, garnered extensive attention from scientists worldwide. The most commonly used heating materials in the atomizing modules of rechargeable e-cigarettes were nickel-chromium alloys, which may have also contained elements such as Fe, Al, V, Mn, Co, Cu, Zn, Ag, and Pb (Jin et al., 2021; Han et al., 2018). These elements were typically detectable in the aerosols produced (Gray et al., 2019; Jităreanu et al., 2022; Rastian et al., 2022). Studies showed that e-cigarette aerosols contained harmful organic chemicals and metals that could cause respiratory problems (Lin et al., 2022). Understanding the composition of e-liquids and heating elements, as well as their interactions, was crucial for analyzing the risk of heavy metal release in e-liquids and aerosols. This knowledge was essential for ensuring the safety of aerosol emissions and protecting consumer health.

As one of the commonly used additives in e-cigarette liquids, sweeteners significantly impacted both the flavor experience and the safety of e-cigarette devices. Rosbrook and colleagues studied the relationship between sucralose, nicotine delivery devices, and the sweetness of e-cigarette flavors, suggesting that the structural design of e-cigarette devices significantly affected the taste experience (Rosbrook et al., 2017) El-Hage and others found that adding 5% sucralose to e-cigarette liquids could produce harmful substances such as 3-monochloro-1,2-propanediol (3-MCPD) and 1,3-dichloropropanol (1,3-DCP) under certain conditions, which were also present in aerosols (El-Hage et al., 2019). Korzun analyzed the thermal degradation products in e-cigarette liquids, detecting a substantial amount of free chloride in aerosol samples, resulting from the decomposition of sucralose (Korzun, 2018). While these studies highlighted the significant effects of sweeteners on the flavor and safety of e-cigarette products, they also exposed a gap in our understanding of how sweeteners influenced the release of heavy metals from atomizers and aerosols, as well as a general shortfall in comprehensive research on metal corrosion and migration within these devices. Due to the extensive scrutiny of sucralose and the rising interest in safer alternatives, our research sought to bridge this gap by comparing sucralose with neotame. Although less studied, neotame was frequently used in e-cigarette liquids and shared chemical similarities with sucralose. However, it was reputed for better heat stability, which may have enhanced its safety profile under the high-temperature conditions typical of vaping devices. Therefore, our study specifically targeted these sweeteners, sucralose and neotame, to evaluate their impacts on the safety of heavy metals in e-cigarette liquids and aerosols. By analyzing both the cytotoxicity and the mechanisms of metal migration, we aimed to provide scientifically grounded insights that could guide the development of safer e-cigarette formulations and ensure consumer safety.

## 2 Materials and methods

### 2.1 Materials and instruments

The base e-liquid used in this study, as well as the e-liquids containing neotame and sucralose sweeteners, were provided by Hongfu Biotechnology (Dongguan, China). The e-liquids were specifically formulated for this experiment, with all samples containing a base e-liquid formula of propylene glycol (PG) and vegetable glycerin (VG) in a ratio of 4:6, to which specified amounts of sweeteners were separately added. The disposable electronic cigarette devices used for aerosol testing were provided by Honeycomb Workshop Electronic Technology (Dongguan, China). The device specifications included an output voltage of 3.6 V, electrical resistance of 1.2 Ω, e-liquid filling volume of 2 mL, and a heating element made of nickel-chromium alloy spring heating wire. The SH-SY-5Y cells used for cytotoxicity experiments were purchased from ICELL (Shanghai, China). The cell culture incubator and biosafety cabinet were provided by Esco Micro Pte (Singapore). The Synergy HT Microplate Reader for cytotoxicity experiments was purchased from BioTek (United States).

The Prisma E scanning electron microscope by Thermo Fisher Scientific (United States) was used to photograph the microscopic morphology of metal surfaces. The Xplore energy dispersive spectrometer (EDS) by Oxford (United Kingdom) was used to determine the elemental composition of materials. The JY-JM06 electronic puffing machine by Jingyao (China) was used for e-cigarette smoking experiments. The Inductively Coupled Plasma Mass Spectrometer, ICP-MS 7850, by Agilent Technologies (United States) was used for heavy metal content detection. The Programmable DC Power Supply IT6800A/B by ITECH (United States) was used for e-liquid heating experiments. The CIC-D120 High Stability Ion Chromatograph by Shenghan (China) was used for chloride ion content detection.

### 2.2 Methods

#### 2.2.1 Determination of heavy metal content in e-liquid

The e-liquid heavy metal test samples were divided into two parts: original e-liquid samples and heated e-liquid samples. The heating of the e-liquid was achieved through a programmable DC power supply connected to multiple sets of nickel-chromium alloy heating meshes, based on the principle of resistance heating. The heating mesh was directly placed into the e-liquid bottle, powered by the DC power supply with a constant voltage output of 3.6 V, heating for 3 s followed by a 15-s pause, and repeating this cycle 200 times. After the heating process, the heating mesh was removed, and the remaining e-liquid in the bottle was divided into two portions. One part was analyzed using ICP-MS for the content of eleven heavy metals: Al, Cr, Fe, Ni, Cu, As, Cd, Sn, Sb, Hg, and Pb; the other part was used for cytotoxicity experiments. The list of e-liquid heavy metal test samples is shown in Table 1, with five groups of original e-liquid samples from A-1 to E-1 and five groups of heated e-liquid samples from A-2 to E-2.

**TABLE 1:** List of e-liquid samples with different types of sweetener content and heating samples.

Sample name	Base e-liquid	Sweetener	Heating material
A-1	PG:VG = 4:6	—	—
B-1	PG:VG = 4:6	Sucralose (2%)	—
C-1	PG:VG = 4:6	Sucralose (5%)	—
D-1	PG:VG = 4:6	Neotame (2%)	—
E-1	PG:VG = 4:6	Neotame (5%)	—
A-2	PG:VG = 4:6	—	Nickel-chromium alloy
B-2	PG:VG = 4:6	Sucralose (2%)	Nickel-chromium alloy
C-2	PG:VG = 4:6	Sucralose (5%)	Nickel-chromium alloy
D-2	PG:VG = 4:6	Neotame (2%)	Nickel-chromium alloy
E-2	PG:VG = 4:6	Neotame (5%)	Nickel-chromium alloy

To detect the heavy metal content in e-liquids, a series of reagents and standard solutions were prepared, including GB/T 6682 grade 1 water, 1.40 g/mL concentrated nitric acid, and 100 mg/L metal standard solutions. Approximately 0.25 g of the e-liquid sample was placed in a 25 mL volumetric flask and diluted to the mark with the diluent, while blank samples were also prepared. Standard working solutions were prepared at different concentrations and diluted to 100 mL. Sample analysis was performed using ICP-MS under the following operating conditions: RF power of 1550 W, nebulizer gas flow rate of 1.06 L/min, auxiliary gas flow rate of 0.90 L/min, plasma gas flow rate of 15.0 L/min, spray chamber temperature of 2°C, helium gas flow rate of 5 mL/min, and peristaltic pump speed of 0.1 rpm. An internal standard solution (0.5 mg/L) was used for calibration to ensure accuracy, and quality control samples were used to verify data reliability. Each set of experiments included four parallel groups to average the results, providing a robust measure of heavy metal concentrations. Standard deviation was calculated for these measurements to serve as error bars, enhancing the reliability and statistical validity of the findings.

#### 2.2.2 Determination of heavy metal content in aerosols

To detect the heavy metal content in aerosols generated during e-cigarette use, we utilized a commercial e-cigarette smoking machine, model JY-JM06, manufactured by Jingyao (China). The specific steps were as follows: disposable e-cigarettes containing e-liquids with different proportions of sweeteners were connected via tubing to an aerosol collection solution containing an acidic matrix (20 mL of 5% nitric acid-2 mg/L gold mixed solution, pH 3–4). The smoking machine generated negative pressure, drawing the aerosol into the collection solution, and then routed it back to the smoking machine through another tube. The smoking machine was set to a puff volume of 55 mL per puff, with each puff lasting 3 s followed by a 27-s interval. Each e-cigarette was subjected to 200 puffs to simulate actual usage. After the puffing process, the collection solution was stored in polyethylene bottles pre-cleaned with ultrapure water and soaked overnight in 10% nitric acid. All samples were stored at 4°C–10°C and subsequently analyzed for heavy metal content using ICP-MS. To ensure statistical robustness, four parallel replicates were performed in each group of experiments.

#### 2.2.3 Cell culture and toxicology experiments

SH-SY-5Y cells and Beas-2B cells were grown in DMEM plus 10% fetal bovine serum (Hyclone, Australia), 100 U/mL penicillin/streptomycin. Cell cultures were maintained at 37°C in a humidified atmosphere containing 5% CO2 and were passed every 2–4 days based on 85% confluence. The cell counting kit-8 assay was used to detect the viability of cells. All of the procedures were followed according to the manufacturer’s instructions. Briefly, cell suspensions (100 μL, 1 × 10 ^4 cells/mL) were added to a 96-well plate and grown overnight. After incubating with samples in 96-well microtiter plates for 24 h, CCK-8 solution (10 μL) was put into the 96-well plate and incubated for 1 h. Finally, absorbance at 450 nm of the wells was measured by the microplate reader, and four replicate wells were assessed in each experiment. The samples used in the cytotoxicity experiments were the heated e-liquid samples from A-2 to E-2.

#### 2.2.4 Analysis of the surface morphology of mesh coil

A Scanning Electron Microscope (SEM) was used to observe the initial microstructure of the mesh coil alloy, and the main components of the alloy were determined using the area scan mode of the SEM’s Energy Dispersive Spectroscopy (EDS) module. After 200 heating cycles in e-liquid samples from A-2 to E-2, we removed the mesh coil samples and placed them in ethanol for ultrasonic cleaning (60 W, 15 min) and drying treatment (120°C, 90 min). Subsequently, we used SEM again to observe the surface corrosion and metal migration phenomena in the five groups of mesh coil samples.

#### 2.2.5 Determination of chloride ion concentration in e-liquid containing sucralose after heating

To determine the possibility of hydrochloric acid production from the decomposition of sucralose in e-liquids upon heating, we measured the concentration of chloride ions in e-liquids containing 5% sucralose after heating at different temperatures using an ion chromatograph. The specific testing method followed the standard procedures outlined in the General Rules for Ion Chromatography Analysis (JY/T 0575-2020). The list of test samples is shown in Table 2.

**TABLE 2:** List of test samples for chloride ion concentration in e-liquid after heating.

Sample name	Base e-liquid	Sweetener	Heating conditions
No.1	PG:VG = 4:6	Sucralose (5%)	Room temperature (24°C)
No.2	PG:VG = 4:6	Sucralose (5%)	150°C, 60 min
No.3	PG:VG = 4:6	Sucralose (5%)	200°C, 60 min

## 3 Results and discussion

### 3.1 Types and concentrations of heavy metals in the original e-liquid

**FIGURE 1:**
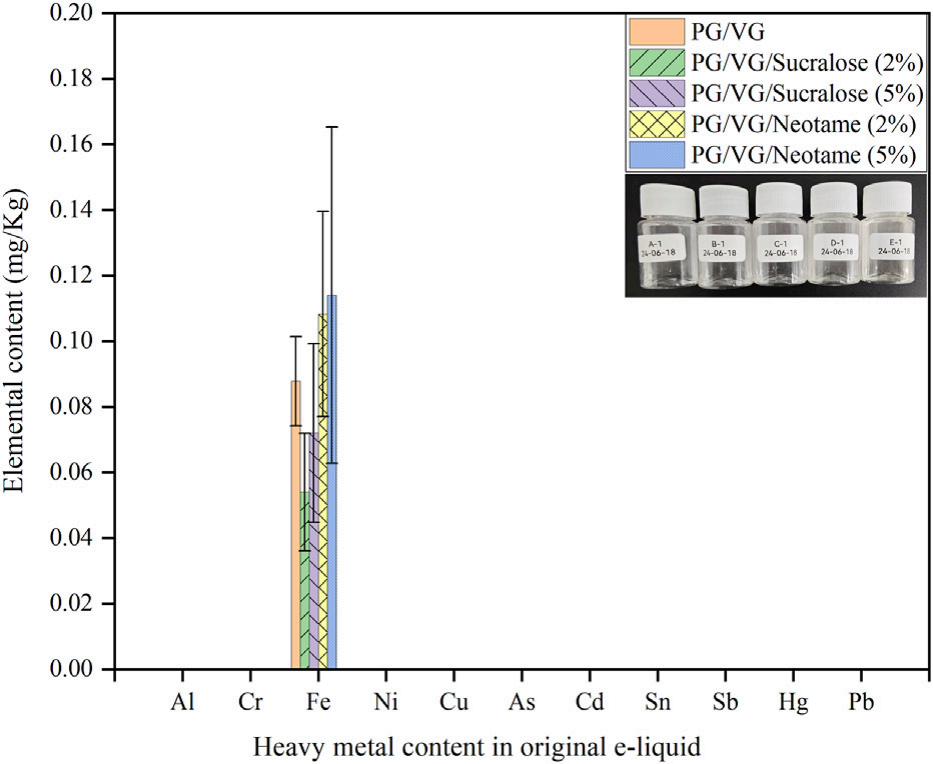
Distribution of heavy metals in original e-liquids containing different sweeteners.

Figure 1 displayed the content of heavy metals in five groups of original e-liquids, as illustrated in the embedded images within the bar chart. Sample A-1 consisted of pure PG/VG e-liquid, while samples B-1 and C-1 contained 2% and 5% Sucralose in PG/VG e-liquid, respectively. Samples D-1 and E-1 contained 2% and 5% Neotame in PG/VG e-liquid, respectively. The analysis of eleven heavy metals (Al, Cr, Fe, Ni, Cu, As, Cd, Sn, Sb, Hg, Pb) in these five groups of e-liquids revealed that the concentration of heavy metals in all samples was low, not exceeding 0.2 mg/kg. Iron was the only metal detected in all e-liquid samples no matter of sweetener concentration. Notably, the Fe content was slightly higher in e-liquids containing Neotame compared to those containing Sucralose and the original e-liquids. All five e-liquid groups contained only iron, likely due to the mandatory mechanical stirring involved in their production process, where contact between the e-liquid and the stainless-steel barrels and mixers could introduce trace amounts of Fe (Palazzolo et al., 2017). The analyses from A-1 to E-1 confirm that apart from trace amounts of Fe, no other heavy metals such as Al, Cr, Ni, Cd, Sb, Hg, Pb, Cu, As, or Sn were present, demonstrating that the original e-liquids are relatively safe and eliminating the impact of inherent heavy metals in the e-liquids for subsequent experiments on e-liquid heating and aerosol heavy metal detection.

### 3.2 Types and concentrations of heavy metals in the heated e-liquid

**FIGURE 2:**
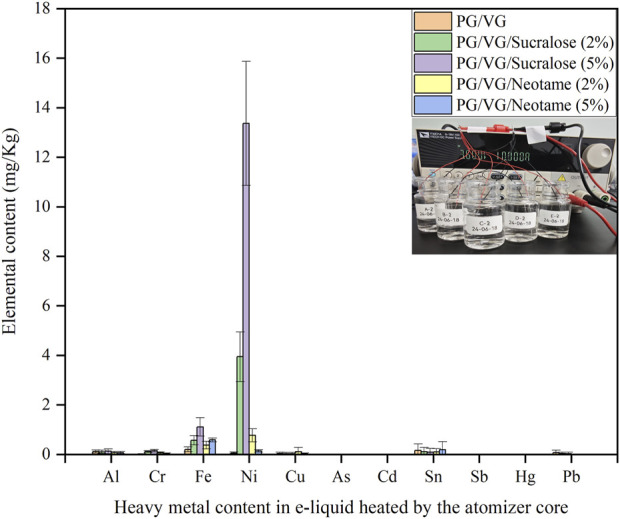
Distribution of heavy metals in e-liquid after heating through a nickel-chromium alloy mesh coil.

Figure 2 showed the distribution of heavy metals in e-liquids after being heated through a nickel-chromium alloy mesh coil, where samples A-2 to E-2 shared the same original e-liquid components as A-1 to E-1. Samples A-2 to E-2 were cyclically heated 200 times through a nickel-chromium alloy mesh coil under a 3.6 V output voltage, as illustrated by the inset diagram in Figure 2, which depicted the heating process by a programmable DC power supply to simulate an e-cigarette atomization. The detection of various heavy metals after heating revealed the presence of Ni, Cr, Fe, Al, Cu, Sn, and Pb to varying extents in the heated e-liquids, with Ni showing the most significant increase, which was clearly associated with the primary composition of the nickel-chromium mesh coil being Ni, Cr, and Fe. Comparing the Ni content in different e-liquids from Figure 1, the highest Ni content was found in the e-liquid containing 5% sucralose at 13.36 ± 2.50 mg/kg, while the e-liquid without sweeteners had the lowest Ni content at 0.02 ± 0.01 mg/kg. E-liquids containing 2% and 5% Neotame showed little difference in Ni content, around 0.1–0.8 mg/kg, which was lower than that in e-liquids with 5% sucralose at 3.94 ± 1.00 mg/kg. Similar patterns were observed for Fe and Cr distributions. The detection of small amounts of Sn, Pb, and Al elements might be attributed to metal impurities introduced during the manufacturing or welding processes of the mesh coil (Omaiye et al., 2021; Palazzolo et al., 2017). Referencing the French AFNOR e-cigarette standards, which stipulate that the Ni content in e-liquids should not exceed 5 mg/kg (Afnor, 2021a), the use of e-liquids with more than 2% sucralose content could result in non-compliance with these guidelines, while using up to 5% Neotame or no sweeteners would not likely lead to significant heavy metal exceedance.

### 3.3 Types and concentrations of heavy metals in aerosols

**FIGURE 3:**
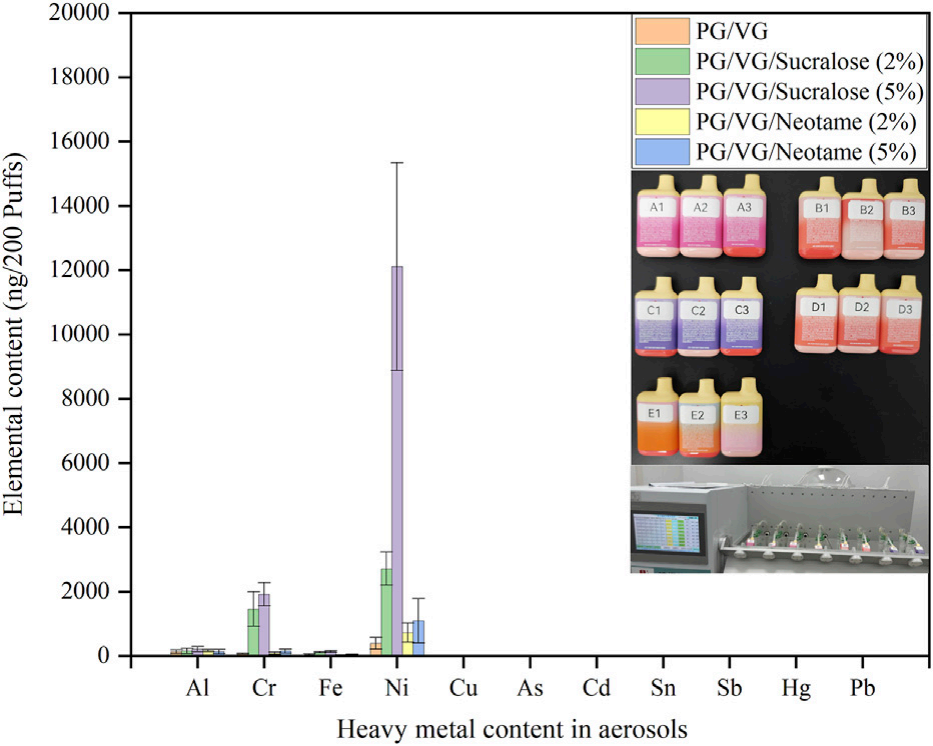
Types and concentrations of heavy metals in aerosols from e-liquids with different sweeteners.

Figure 3 illustrated the average concentrations of heavy metals captured in aerosols after 200 cycles of puffing for 3 s with a 27-s pause on e-cigarettes filled with e-liquids containing sweeteners of different types and concentrations. The e-liquids used in samples A to E were the same as those in A-1 to E-1. The morphology of the e-cigarette samples and the experimental process were illustrated in the inset of Figure 3, and all samples were tested under consistent device and puffing conditions. From the results of the heavy metal tests, it was found that the e-cigarette atomizer had converted the e-liquids into aerosols containing elements such as Ni, Cr, Fe, and Al. Notably, sample C, which contained 5% sucralose, had the highest Ni content in the aerosols (12,109 ± 3,229 ng/200 puffs), consistent with the highest Ni content in the sucralose-containing e-liquids shown in Figure 2, evidently related to the Ni composition of the mesh coil. Comparing the concentrations of Ni, Cr, and Fe in the aerosols of different samples, a pattern emerged where B (2,717 ± 515 ng/200 puffs) and C were higher than D (723 ± 299 ng/200 puffs) and E (1,094 ± 692 ng/200 puffs), with B being less than C, and D less than E. This indicated that the content of sucralose in the e-liquids significantly affected the aggregation of heavy metals in the e-cigarette aerosols, with higher sucralose content leading to increased aggregation of Ni, Cr, and Fe elements. In contrast, neotame did not show a consistent pattern in influencing the precipitation of these heavy metals in the e-liquids, and the precipitation amounts were significantly lower compared to those with the same concentration of sucralose. E-liquids containing Neotame and those without any sweeteners showed no significant difference in heavy metal content in the aerosols. Referencing the French AFNOR e-cigarette standard, which stipulates that the Ni content in aerosols should not exceed 5,000 ng/200 puffs (Afnor, 2021b), the use of e-liquids containing more than 2% sucralose for atomization may lead to exceeding these recommended limits, while e-liquids with up to 5% Neotame or no sweeteners do not exhibit significant exceedance of Ni in the aerosols. The data above indicate that compared to sucralose, using neotame as an additive in e-cigarette e-liquids is safer, effectively reducing the content of Ni, Cr, Fe, and Al metals in aerosols.

### 3.4 Cytotoxicity assessment of different e-liquids after heating by the mesh coil

**FIGURE 4:**
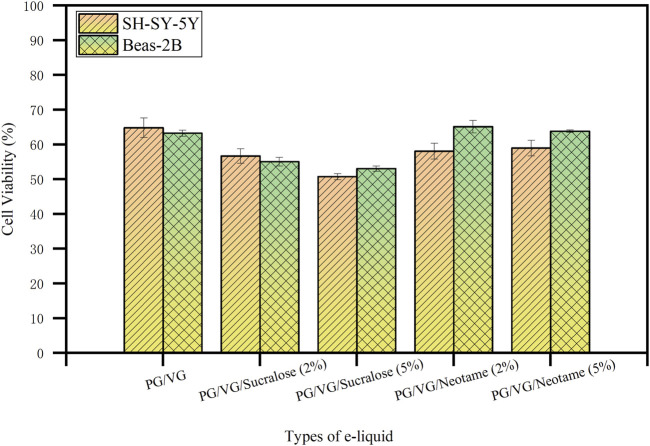
The effect of e-liquids with different sweeteners and concentrations on the viability of SH-SY-5Y and Beas-2B cells after heating.

As shown in Figure 4, the effect of e-liquids with different concentrations of sucralose and neotame on the viability of SH-SY-5Y neuroblastoma cells and Beas-2B bronchial epithelial cells was investigated. The experiments employed five different e-liquid formulations heated using mesh coils. Cell viability was assessed using the CCK-8 assay, which revealed a decrease associated with increasing concentrations of sucralose. Specifically, cell viability for SH-SY-5Y was 64.80% ± 2.84% with the base PG/VG e-liquid (A-2), 56.66% ± 2.11% with 2% sucralose (B-2), and had dropped to 50.74% ± 0.88% with 5% sucralose (C-2). In contrast, e-liquids containing neotame showed relatively less cytotoxicity; viability was 58.06% ± 2.26% with 2% neotame (D-2) and 58.96% ± 2.23% with 5% neotame (E-2). Similar trends were observed in Beas-2B cells, where viability was 63.24% ± 0.86% for the base e-liquid, 55.04% ± 1.24% for 2% sucralose, 53.03% ± 0.76% with 5% sucralose, 65.10% ± 1.78% with 2% neotame, and 63.80% ± 0.35% with 5% neotame. These results underscore the enhanced cytotoxic potential of sucralose at higher concentrations, potentially linked to increased heavy metal release under heating conditions, which correlates directly with its cytotoxic effects (Chafin, 2022; Eshraghian and Al-Delaimy, 2021)^.^ Neotame’s lower impact on cytotoxicity suggests its stability and less propensity to release heavy metals when heated. In conclusion, using high concentrations of sucralose as a sweetener in e-cigarette products may pose safety risks due to the increased release of heavy metals. In contrast, neotame as a sweetener is less likely to cause excessive heavy metal release and is relatively safer.

### 3.5 Corrosion of the mesh coil surface by e-liquid

**FIGURE 5:**
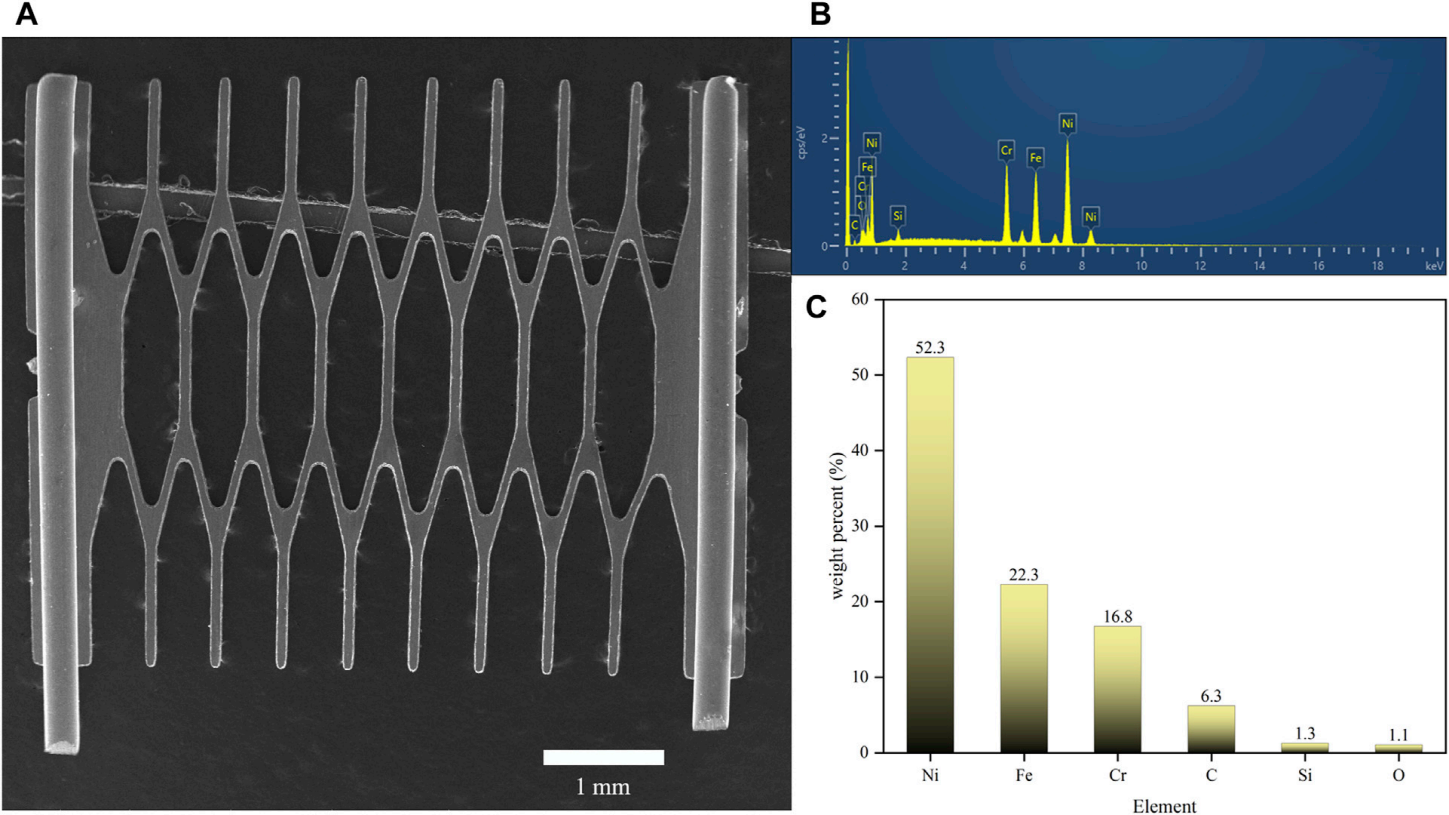
Surface morphology and material composition analysis of the mesh coil. **(A)** SEM surface morphology image of the mesh coil. **(B)** Energy spectrum analysis chart of the mesh coil material. **(C)** Elemental composition percentage of the mesh coil.

To investigate the mechanism by which different sweeteners influenced the leaching of heavy metals such as Ni, Cr, and Fe from mesh coils into e-liquids, we first analyzed the surface morphology and material composition of the mesh coils using SEM and EDS, as shown in Figure 5. Figure 5A presented the overall morphology of the mesh coil, with cylindrical pins at both ends and a diamond-shaped mesh structure in the middle representing the heating zone. The e-liquid boiled and vaporized upon contact with this high-temperature surface. The EDS spectrum in Figure 5B indicated that the primary elements in the heating zone of the mesh coil were Ni, Cr, and Fe, while C, Si, and O, common contaminants in EDS analysis, could be ignored (Postek, 1996). As shown in Figure 5C, the composition of the alloy in the mesh coil is 52.3% Ni, 22.3% Cr, and 16.8% Fe, with Ni having the highest concentration. This correlates with the higher Ni content detected in both the e-liquid and aerosol heavy metal analyses.

**FIGURE 6:**
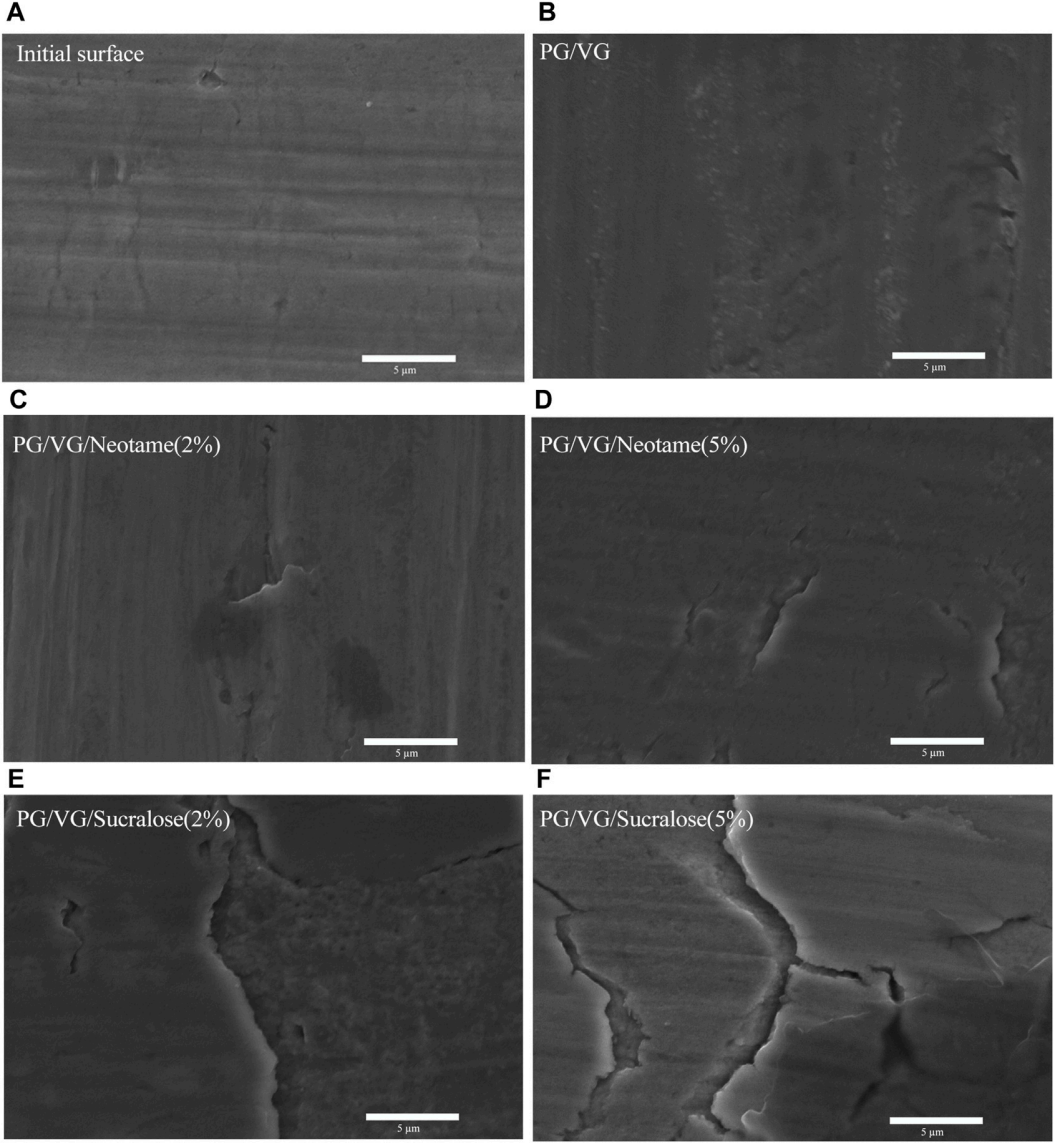
SEM images of the mesh coil surface after heating different e-liquids. **(A)** Surface of the original mesh coil without heating, **(B)** Heated with pure PG/VG e-liquid, **(C)** PG/VG/Neotame (2%), **(D)** PG/VG/Neotame (5%), **(E)** PG/VG/Sucralose (2%), **(F)** PG/VG/Sucralose (5%).

The micrographs of the original and post-heating surface morphologies of the mesh coil in contact with different e-liquids are presented in Figure 6. Figure 6A reveals that the original surface of the mesh coil is not entirely smooth, displaying slight cracks and defects likely caused by the stamping process during coil formation, which can induce localized compression and bending. Figure 6B shows that after cyclic heating in pure PG/VG e-liquid, slight metal migration and accumulation occur. Figures 6C–F illustrate the surface morphology after heating in contact with e-liquids containing sweeteners. The surface of the mesh coil exhibits significant cracking and metal layer delamination after cyclic heating with sucralose-containing e-liquid. In contrast, when in contact with neotame-containing e-liquid, the coil shows localized defect corrosion, though much less severe than with sucralose. Considering the differences in heavy metal content (Ni, Cr, Fe) in various e-liquids shown in Figure 2, it becomes evident that the primary source of heavy metals leaching into the e-liquids is the migration and delamination of metals from the mesh coil surface. Sucralose-containing e-liquids significantly exacerbate this metal corrosion, migration, and delamination.

### 3.6 Analysis of chloride ion concentration in sucralose-containing e-liquid after heating

**FIGURE 7:**
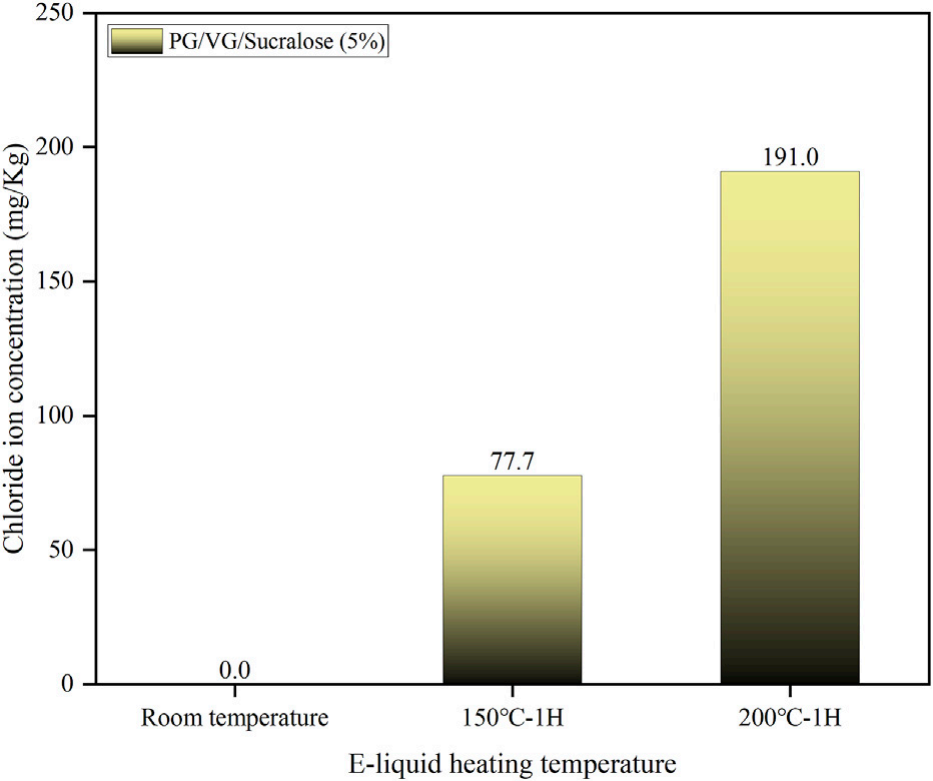
The concentration of chloride ions in sucralose-containing e-liquids at different temperatures.

The heating process of e-liquid containing sucralose causes severe corrosion on the surface of Ni-Cr mesh coils. To confirm the cause of this corrosion, we analyzed the concentration of chloride ions in e-liquid containing 5% sucralose after heating at room temperature, 150°C, and 200°C for 1 h, based on the chemical property of sucralose decomposing to release chloride ions upon heating (Duell et al., 2019; Eisenreich et al., 2020). As shown in Figure 7, no chloride ions were detected in the e-liquid at room temperature. However, after heating at 150°C for 1 h, the chloride ion concentration in the sample reached 77 mg/kg, and after heating at 200°C for 1 h, the concentration increased to 191 mg/kg. Typically, the operating temperature of an e-cigarette atomizer during vaping ranges between 220°C and 300°C. Combined with the corrosion images of the mesh coil surface shown in Figure 6F, it is evident that sucralose in the e-liquid decomposes under the high temperatures during the atomizer’s operation, producing a large amount of chloride ions. These ions engage in electrochemical reactions with the metal surface, causing metal ions to leach into the solution and form metal chlorides, such as nickel chloride and chromium chloride. The formation of these chlorides leads to localized corrosion of the Ni-Cr alloy surface, particularly in areas with microcracks or defects. Therefore, in e-cigarette products, when the e-liquid contains a high concentration of sucralose, the continuous cyclic heating can lead to an increasing concentration of chloride ions in the e-liquid. This results in ongoing corrosion of the metal parts in contact with the e-liquid, potentially migrating into the e-liquid and compromising user safety.

## 4 Conclusion

This study investigated the impact of heating e-liquids with different concentrations of sucralose and neotame on heavy metal release in e-liquids and aerosols, as well as their cytotoxicity. The results showed that e-liquids containing 5% sucralose had the highest heavy metal release, especially nickel (13.36 ± 2.50 mg/kg in e-liquids and 12,109 ± 3,229 ng/200 puffs in aerosols), while e-liquids containing neotame had significantly lower heavy metal content. Cytotoxicity experiments demonstrated that among both SH-SY-5Y neuroblastoma cells and Beas-2B bronchial epithelial cells, the base e-liquid (PG/VG) maintained the highest cell viability after heating, with values of 64.80% ± 2.84% and 63.24% ± 0.86%, respectively. Conversely, e-liquids containing 2% and 5% sucralose significantly reduced cell viability to 56.66% ± 2.11% and 50.74% ± 0.88% in SH-SY-5Y cells, and to 55.04% ± 1.24% and 53.03% ± 0.76% in Beas-2B cells, respectively. SEM analysis showed significant corrosion and cracking on the mesh coil surface after heating with sucralose-containing e-liquids. In contrast, neotame had minimal impact on the metal surface morphology, indicating its less corrosive effects compared to sucralose. This was related to the release of chloride ions from sucralose at high temperatures, leading to hydrochloric acid formation, which caused metal corrosion and heavy metal release. E-liquids with neotame showed less corrosion, indicating that neotame is relatively stable during heating with a lower risk of heavy metal release. In conclusion, e-liquids with high concentrations of sucralose result in higher heavy metal release and greater cytotoxicity, suggesting that the sucralose content in e-liquids should be limited to 2% to reduce potential health risks. Neotame, on the other hand, exhibited lower heavy metal release and cytotoxicity, making it a safer sweetener choice for e-liquid formulations.

## Data Availability

The original contributions presented in the study are included in the article/supplementary material, further inquiries can be directed to the corresponding author.
